# Effects of maternal inhalation of carbon black nanoparticles on reproductive and fertility parameters in a four-generation study of male mice

**DOI:** 10.1186/s12989-019-0295-3

**Published:** 2019-03-18

**Authors:** Astrid Skovmand, Alexander C. Ø. Jensen, Clotilde Maurice, Francesco Marchetti, Anna J. Lauvås, Ismo K. Koponen, Keld A. Jensen, Sandra Goericke-Pesch, Ulla Vogel, Karin S. Hougaard

**Affiliations:** 1 The National Research Centre for the Working Environment, Copenhagen Ø, Denmark; 2 Department of Veterinary Clinical Sciences, University of Copenhagen, Frederiksberg C, Denmark; 3 Institute of Public Health, University of Copenhagen, Copenhagen K, Denmark; 4 Department of Micro- and Nanotechnology, Technical University of Denmark, Lyngby, Denmark; 5 Reproductive Unit of the Clinics – Clinic for Small Animals, University of Veterinary Medicine Hannover, Foundation, Hannover, Germany; 6 Environmental Health Science Research Bureau, Health Canada, Ottawa, ON Canada

**Keywords:** Computer assisted sperm analysis, Daily sperm production, In utero, Nanoparticles, Reproductive toxicity, Airway exposure, Sperm quality, Testes

## Abstract

**Background:**

Previous findings indicate that in utero exposure to nanoparticles may affect the reproductive system in male offspring. Effects such as decreased sperm counts and testicular structural changes in F1 males have been reported following maternal airway exposure to carbon black during gestation. In addition, a previous study in our laboratory suggested that the effects of in utero exposure of nanoparticles may span further than the first generation, as sperm content per gram of testis was significantly lowered in F2 males. In the present study we assessed male fertility parameters following in utero inhalation exposure to carbon black in four generations of mice.

**Results:**

Filter measurements demonstrated that the time-mated females were exposed to a mean total suspended particle mass concentration of 4.79 ± 1.86 or 33.87 ± 14.77 mg/m^3^ for the low and high exposure, respectively. The control exposure was below the detection limit (LOD 0.08 mg/m^3^). Exposure did not affect gestation and litter parameters in any generation. No significant changes were observed in body and reproductive organ weights, epididymal sperm parameters, daily sperm production, plasma testosterone or fertility.

**Conclusion:**

In utero exposure to carbon black nanoparticles, at occupationally relevant exposure levels, via maternal whole body inhalation did not affect male-specific reproductive, fertility and litter parameters in four generations of mice.

## Background

Epidemiological research shows that semen quality across the globe has and continues to decline. A statistical analysis performed in 1992 based on data derived from world literature shows that from 1940 to 1990 the mean sperm counts of fertile men declined from 113 × 10^6^ to 66 × 10^6^ sperm/ml [1]. A recent analysis (2017) continues to support this statement and shows that from 1973 to 2011 sperm counts have declined 50–60% in North America, Europe, Australia and New Zealand [2]. In countries such as Denmark, young cohorts with sperm counts in the subfertility range also have lowered fecundity, suggesting that poor semen quality is a potential contributing factor to low fertility rates [3]. Some recent data, however, indicate that sperm counts have stabilized in at least some countries [4]. The decline in sperm numbers may be caused by the sensitivity of the male reproductive system to a number of occupational and environmental toxicants [5]. Likewise, maternal exposure to environmental hazards during critical windows of development may negatively impact the fetus and lead to health problems later in life, including reduced semen quality and reproductive dysfunction in the male offspring [6–10].

Particulates have been acknowledged as potential health risks through occupational, consumer and environmental exposures. Maternal airway exposure to carbonaceous nanoparticles (NPs) during gestation has been shown to cause changes in the male reproductive system in the first (F1) and second generation (F2). Exposure to 20 mg/m^3^ of diesel exhaust particle (SRM2975) on gestation days 7 to 19 via whole body inhalation, for example, reduced daily sperm production (DSP) in the F1 offspring [11]. Likewise, intratracheal instillation of 0.2 mg/mouse of 14 nm carbon black (CB) on gestation day 7 and 14 caused testicular structural changes and reduced DSP in the F1 offspring [12]. Intergenerational effects on DSP have also been observed following intratracheal instillation of 67 μg of Printex 90 on gestation days 7, 10, 15 and 18 [13]. Although sperm content per gram of testis was unaffected in the F1 males, their offspring (F2) had reduced sperm numbers [13]. The effects on the F2 offspring may be due to direct in utero exposure, as both the F1 offspring and the F1 developing germ cells, i.e., the F2, would have been exposed [14]. Thus, investigations of transgenerational effects require the analysis of at least the third generation (F3).

Inhaled NPs deposit primarily in the alveolar region in the lungs from where they may translocate into the systemic circulation. For example, CB Printex 90 NPs have been shown to translocate to the liver following pulmonary exposure [15]. Animal studies and in vitro cellular barrier models have demonstrated that NPs in circulation can potentially cross the placenta, moving from the maternal to the fetal compartment [16–18]. Insoluble NPs induce inflammation which is determined by the total deposited surface area. In addition, some NPs including CB are strong generators of reactive oxygen species (ROS) [19]. Fetal development may be disrupted by oxidative stress as well as by inflammatory mediators [16]. Furthermore, in females, inflammatory mediators may interfere with the neuroendocrine axis and disrupt normal gonadotropic hormone secretion from the anterior pituitary gland; maternal hormone homeostasis is crucial for normal fetal development, particularly of the male reproductive system [20, 21].

Previous in utero exposure studies found effects on the male reproductive system (i.e. reduced testicular sperm counts) in offspring from pregnant mice exposed to CB particles by intratracheal instillation. Here, we hypothesized that similar effects would be induced when using the most relevant route of exposure in humans, i.e., inhalation, at occupationally relevant exposure levels. This study aimed to investigate the transgenerational effects from the F1 to the fourth generation (F4), on reproductive function of male offspring originating from time-mated female mice exposed by whole body inhalation to 4.6 mg/m^3^ and 37 mg/m^3^ of Printex 90 for 45 min/day from gestation day 4 to 18.

## Methods

### Animals, parturition and lactation

All experimental procedures followed the handling guidelines established by the Danish government and permits from the Experimental Animal Inspectorate (no. 2015–15–0201-00569). Prior to the study, the specific experimental protocols were approved by the local Animal Ethics Council. Sixty time-mated outbred NMRI mice were purchased from Taconic Biosciences Inc. (Ejby, Denmark). Briefly, pregnant females arrived at the facility on gestation day 3 and were semi-randomised into three groups of 20 animals with equal weight distribution and housed in groups of five. The exposure began on gestation day 4 and, after the last exposure on gestation day 18, the females were transferred to single housing. The day of the delivery was designated postnatal day (PND) 0 and F1 pups were counted and sexed on PND 1. Dams and individual pups were weighed at PND 1, 8, 12 and 17. Out of the 20 exposed dams, 6, 4 and 5 dams of the control, low and high dose, respectively, did not produce a litter. The non-pregnant time-mated females were euthanized on PND 9 (i.e., 11 days post exposure) and subjected to bronchoalveolar lavage (BAL). The remaining dams (delivering litters) were euthanized after weaning on PND 26–27 (i.e., 28–29 days post exposure). BAL was followed by determination of uterine implantation sites and losses.

**Fig. 1:**
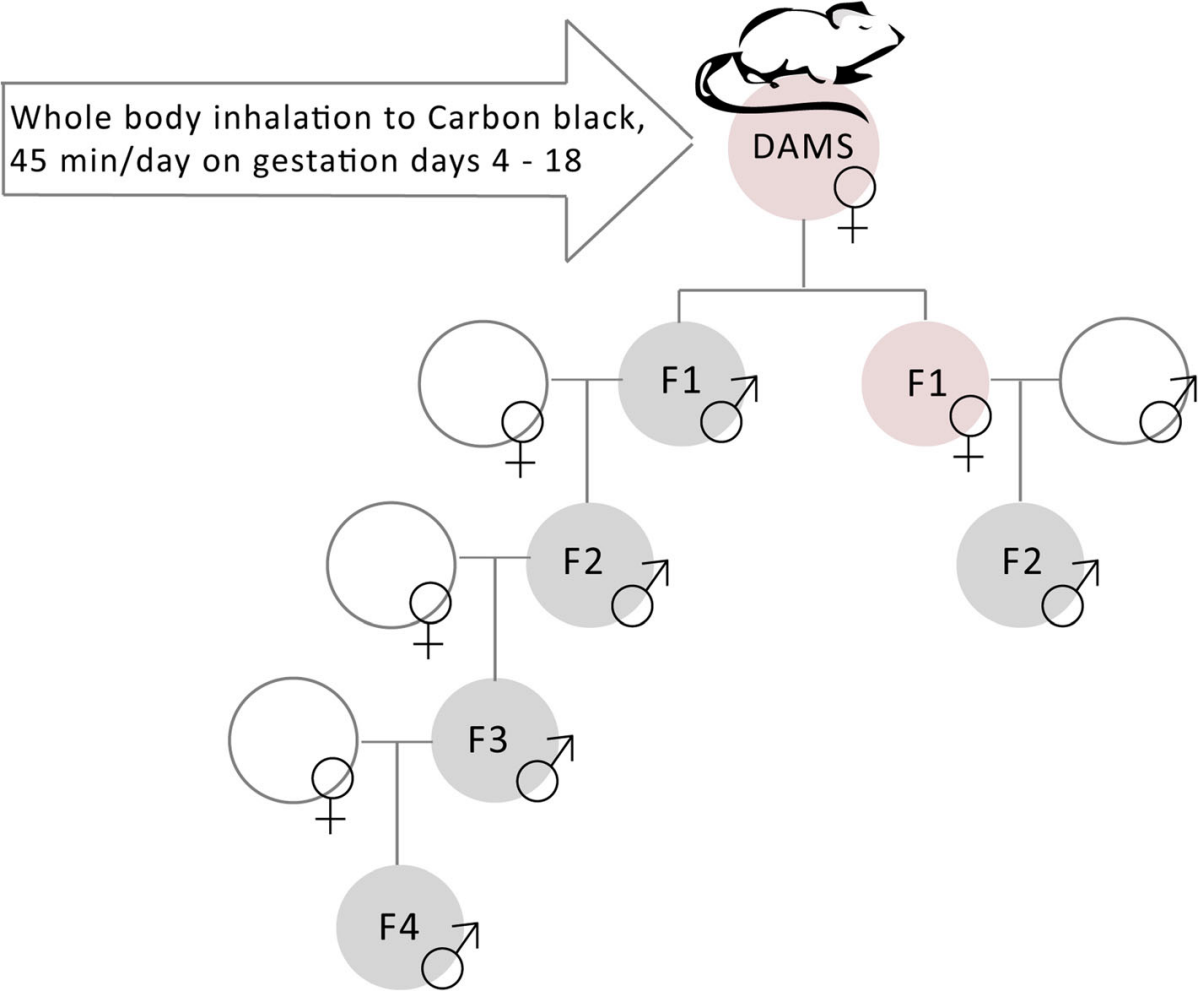
Time mated female NMRI were exposed via whole body inhalation to 4.6 and 37 mg/m^3^ of CB Printex 90 or HEPA-filtered clean air for 45 min a day from gestation day 4 to 18. The male germ line was mated with naïve females for four generations. The female germ line was mated with naïve males for 2 generations. For the fourth generation, only offspring from the clean air and the high dose (37 mg/m^3^) groups were analysed

Both male and female pups from the dam remained with their dams until weaning at PND 21. F1 males and females, 17 weeks of age, were mated with naïve, (non-exposed) sexually mature NMRI females and males, respectively, to produce F2 males and F2 females of the male and female germline, respectively. Only the F2 males derived from F1 males (male germline) were used to produce generation F3 and subsequently F4, see Fig. 1, where mating was initiated at approximately 10 and 11 weeks of age, respectively. For the F2 and F3, dams and offspring were weighed, and litter size and sex ratio determined at PND 2. For logistical reasons, only the control and high dose groups were bred for the F4. Overall, a maximum of one male offspring per litter was used to generate the subsequent generation and assessed for any male reproductive parameters to avoid litter effects. However, to ensure enough males for the breeding of subsequent generations, two F1 males were bred with a naïve female from some litters. During the statistical analysis (see below), reproductive parameters were averaged between males from litters originating from the same female in the parental generation.

Pregnant females were provided tap water and diet (Altromin no. 1314, for breeding, Brogaarden, Denmark) ad libitum. Adult male offspring were housed in groups of five and had access to tap water and standard pellet diet (Altromin no. 1324, Brogaarden, Denmark) ad libitum. All mice were housed in clear 1290D euro standard type 3 cages with aspen sawdust bedding (Tapvei, Estonia), enrichment (mouse house 80-ACRE011, Techniplast, Italy; small aspen blocks, Tapvei, Estonia) and nesting material (Enviro Dri, Lillico, Biotechnology, UK). Housing conditions were kept constant, with a 12:12 h light and dark cycle, an average temperature of 22 °C and 55 ± 10% humidity. The experimental design is described in more detail in Umezawa et al. 2018 [22].

The adult male offspring were euthanized by withdrawal of heart blood at the age of 21, 11, 12 and 8 weeks for the F1, F2, F3 and F4, respectively, following intraperitoneal injection of a cocktail of Zoletil Forte 250 mg/ml, Rompun 20 mg/ml, Fentanyl 50 μg/ml in sterile isotone saline, at the total volume of 0.1 ml/10 g body weight. Reproductive organs were weighed during collection. The left testes were fixed in Bouin’s solution for histological analysis of paraffin-embedded tissue and the right testes were frozen in liquid nitrogen. The left epididymes were used for sperm collection and the right epididymes were frozen in liquid nitrogen. Frozen tissue was stored at − 80 °C. Samples for analysis of plasma testosterone, DSP and for sperm chromatin structure assay (SCSA) were blinded prior to analysis.

### Exposure to carbon black nanoparticles

The CB Printex 90 was a gift from Degussa-Hüls (Frankfurt, Germany). It is a spherical particle with a primary particle size of 14 nm and a specific surface area of 183 m^2^/g with negligible contents of PAH and endotoxin [23, 24]. Pregnant females were placed separately in a cylindrical wire mesh cage (diameter 29 cm, height 9 cm) divided radially into smaller triangular spaces holding up to 12 animals at a time. The cage was placed in an 18 L spheroidal chamber with a stainless steel rim and upper and lower spheres in fully transparent pyrex glass designed to provide an evenly distributed exposure atmosphere. Printex 90 was aerosolized at a pressure of 5 bar using a venturi-type rotating disc microfeeder (Fraunhofer Institute für Toxicologie und Aerosolforschung, Hannover, Germany) and directed through an aerosol mixing and sedimentation glass-tube to the exposure chamber at a total volume-flow of 20 L/min. At the lowest rotation setting, the concentration was too high compared to the target exposure, therefore, for the low exposure, the CB source from the Fraunhofer dispenser was active for 15 min followed by 7.5 min of inactivity. For the high exposure the dispenser could be run as normal to reach the target high exposure concentration. This sequence was repeated twice for a total exposure time of 45 min/day by whole body inhalation from gestation day 4 to 18, to either a HEPA-filtered clean air (control group) or to target Printex 90 concentrations of 4.6 mg/m^3^ for the low dose and 37 mg/m^3^ for the high dose. The concentration achieved of 4.6 and 37 mg/m^3^ for 45 min per day corresponds to 1 and 8 h, respectively, at the 8 h time weighted average occupational exposure limit of 3.5 mg/m^3^ in Denmark [25, 26].

Mass concentrations of total suspended dust were controlled by filter sampling and were used to adjust and maintain targeted concentrations in the chamber, by sampling continuously on Millipore Fluoropore filters (Diameter 2.5 cm; pore size 0.45 μm) at an air flow of 2 L/min, using Millipore cassettes. Acclimatized filters were pre-weighed on a Sartorius Microscale (Type M3P 000 V001). Final gravimetric data were obtained from acclimatized filters (50%RH and 20 °C). Particle number and aerodynamic particle size distributions in the exposure atmosphere were measured in 14 size channels between 6 nm and 10 μm with 1 s intervals using an Electrical Low Pressure Impactor (ELPI+, Dekati Ltd., Tampere, Finland). Controls were monitored using a condensation particle counter (CPC; model 3007, TSI Inc., Shoreview, MN, USA) with a detection range from 10 nm to > 1 μm. Methods and results on the exposure characterization are also reported in Umezawa et al. 2018 [22].

The deposited dose was estimated using the multiple-path particle dosimetry (MPPD) model [27, 28] using the default values for BALB/c mice. Deposited mass in the head, tracheobronchial, or pulmonary region was estimated according to the equation:

$$ {m}_{dep,j}=V\sum \limits_{i=1}^n{C}_i{D}_{f,i,j} $$

Where j is either head, tracheobronchial, or pulmonary region, i is the channel number of the ELPI, D_f_ is the deposition fraction from the MPPD model corresponding to the logarithmic midpoint of the ELPI channel, C is the converted mass concentration at particle density of 1 g cm^− 3^, and V is the total volume inhaled during exposure in the chamber assuming an inspiration rate of 0.06 L min^− 1^ [29]. The deposited dose in mg/kg was calculated using the average weight for BALB/c mice (~ 20 g).

### Bronchoalveolar lavage fluid and serum amyloid A3 gene expression in lungs

Lung inflammation in the dams was assessed by differential cell counts of BAL fluid 11 (for time-mated females without litters) and 28–29 days post-exposure (for dams with litters). In brief, the lungs were flushed twice with 0.8 ml of 0.9% saline. Total number of cells was determined by a NucleoCounter NC-200TM (Chemometec, Denmark). Cells were centrifuged in a Cytofuge 2 (StatSpin, TRIOLAB, Brøndby, Denmark) and stained with May-Grünwald-Giemsa, blinded, and 200 cells/sample were counted under light microscope. During processing for the differential cell counts, some slides contained less than 200 cells and were therefore excluded from the analysis, resulting in *n* = 4–13. For the serum amyloid A 3 (*Saa3)* analysis, RNA was isolated from the lung tissue on Maxwell® 16 (Promega, USA) using Maxwell® 16 LEV simply RNA Tissue Kit (AS1280, Promega, USA) according to the manufacturer’s protocol. *Saa3* mRNA levels were determined using real-time RT-PCR in triplicates, and were normalised to 18S. Methods and results regarding BAL fluid cell counts and *Saa3* expression in the lungs have also been reported in Umezawa et al. 2018 [22].

### Collection of epididymal sperm and computer-assisted sperm analysis (CASA) of concentration and motility

Epididymal sperm were collected and analysed as previously described in Skovmand et al. 2018 [30]. The left epididymal cauda was placed in 500 μl of warm (37 °C) TCM199 medium (Sigma-Aldrich, Denmark) and minced with scissors. Sperm cells were allowed to swim out for 10 min and then filtered through a stainless steel mesh. Samples were kept at 37 °C on a heating stage during the whole procedure including the microscopy analysis. CASA was performed using a negative phase contrast microscope (Olympus BX60, Tokyo, Japan) equipped with a heating stage and a high-speed GigE camera (avA21000-100gc) with a CCD sensor (aviator series, Basler, Germany) detecting 101 frames/sec and the AndroVision software (Ref 12,500/0000, Software Version 1.0.0.9, Minitube, Tiefenbach, Germany). For analysis of concentration and motility, an aliquot of the semen (2.0 μl) was pipetted into an evaluation chamber (Leja® Standard Count 4 Chamber Slide, 10 μm, Leja Products B.V., Nieuw Vennep, The Netherlands) and 10 randomly distributed fields were analysed at 200x magnification. The software calculated the number of sperm per ml and analysed sperm motility parameters: total motility, progressive motility, rapid motility, slow motility, circular motility and local motility. The results presented are the percentage of total motility and progressive motility. The following settings on the CASA system were used: sperm recognition area 10–100 μm^2^, 10 fields per sample, TM = PM + LM, PM = slow motility + fast motility, LM: velocity curved line (VCL) < 80 μm/s and velocity straight line (VSL) < 20 μm/s, Circular Motility: linearity < 0.6000 and rotation > 0.8000.

### Daily sperm production

The adipose tissue from the frozen testes was trimmed off and the tunica albuginea removed by first making a shallow longitudinal incision and then peeled away with forceps. The testes were weighed and placed into 8 ml of 0.05% TRITON-X100 and homogenized for 3 min using the IKAULTRA TURRAX T25 disperser S25 N-10G. Homogenates were kept on ice for 30 min. 200 μl of the homogenate were mixed with 200 μl of 0.04% Trypan blue and left for 5 min at room temperature. Sperm samples were counted in triplicates using an improved Neubauer counting chamber, where the entire centre grid was counted. If the counts in the triple determinations deviated by more than 20%, the procedure was repeated for the sample. DSP was calculated as: **DSP = N / 4.84.** Where N is the total number of spermatids per sample (i.e., sperm number per μl x volume lysis buffer) and 4.84 is the number of days for a spermatid to develop through stages 14 to 16, i.e., the stages where spermatids are resistant to homogenization [30].

### Sperm chromatin structure assay

The F1 epididymal sperm samples remaining from the CASA analysis were frozen, stored at − 80 °C and shipped to Health Canada (Ottawa ON, Canada) on dry ice. Sperm chromatin structure assay (SCSA) was performed as described in Maurice et al. 2018 [31]. Briefly, sperm samples were thawed for 2 min at 37 °C and diluted with Tris Sodium Ethylenediaminetetraacetic acid (TNE) buffer: 0.15 M NaCl, 1 mM EDTA, 0.01 M Tris-HCl, pH 7.4) to get 2 million spermatozoa/ml in a volume of 500 μl. Controls and samples were diluted with the same volume of TNE buffer. Each sample was then mixed with 400 μl of denaturation buffer (0.01 N HCl, 0.15 M NaCl and 0.1% triton X-100, pH 1.4) for 30 s at 4 °C to denature uncondensed sperm DNA. After 30 s, 1.2 ml of acridine orange staining solution (0.126 M Na_2_HPO_4_, 0.037 M citric acid buffer, 1 mM EDTA, 0.15 M NaCl, pH 6.0, containing 6 μg/ml Acridine Orange) were added. Exactly 3 min after the addition of the denaturation buffer, each sample was analysed with a FACSCalibur (BD Biosciences, Mississauga, ON, Canada) fitted with an argon ion laser (488-nm line excitation). A positive control (sperm from a pool of two untreated mice, collected in the same way as the experimental samples) was obtained by pre-incubating the sperm with 20 mM H_2_O_2_ and 10 mM of dithiotheitol (DTT) for 30 min at 37 °C. A positive control sample was run before the first experimental sample and after every 10 samples to monitor instrument stability. A negative control sample (sperm from a pool of three untreated mice, collected in the same way as the experimental samples) without treatment was run at the beginning to set up flow cytometer parameters. A minimum of 10,000 sperm were analysed per sample. Raw data were analysed using WinList Software (Verity Softaware House, Topsham, ME). Results from the SCSA are presented as DNA fragmentation index (%). DFI was calculated as: DFI = red fluorescence/total (red +green) fluorescence.

### Plasma testosterone

Blood was collected from the heart, stabilized using K_2_EDTA and then centrifuged at 2500 g for 10 min. The EDTA-plasma was pipetted into separate snapstrip PCR-vials and stored at − 80 °C until analysis. The levels of testosterone were determined in duplicates and 1:2 dilutions with phosphate-buffered saline (PBS), using competitive ELISA (RTC001R, Biovendor, Brno, Czech Republic) that was validated previously in our lab. Samples were analysed following the manufacturer’s protocol, with a standard curve in the range of 0.1–25 ng/mL. All samples that fell outside the standard curve were diluted 1:4 in PBS. (Interassay) coefficient of variance was 4.8–7.8% [30].

### Statistical analysis

The litter was considered the statistical unit and the number of exposed dams and the number of pups per generation are presented in Table 1. Data were tested for normal distribution using a Shapiro-Wilk normality test. One way ANOVA was used to test for the overall significance of normally distributed data. ANCOVA controlled for litter size in the analyses of maternal and pre-weaning pup weights. For breeding of the F2-generation, where both F1 males and females were mated, PND 2 weights were analysed by a 2-way ANCOVA, with exposure and germline (male/female) as factors. The Kruskal-Wallis test was applied in analysis of gestational parameters, and testosterone concentrations. The latter was presented as geometric mean and geometric standard deviation (SD). Data were analysed using Origin Pro, version 2016 (64-bit), OriginLab Corp (Northampton, MA, USA) and SYSTAT Software Package 9 (California, USA). An a priori power analysis indicated that a group size of 15 would provide a 95% chance of detecting approximately a one-fold difference in the second generation at the 0.05 significance level. The power analysis was performed using G*Power software version 3.1.9.2, (Dusseldorf, Germany).

**Table 1:** Sample sizes for exposure of time-mated dams, pulmonary inflammation and reproductive parameters of male offspring

	Control	Low	High
F0			
Exposed time-mated females	20	20	20
F0			
Dams delivering litters	14	16	15
F0			
BAL fluid cells			
Non-littering females	4	4	4
Littering females	5	9	13
F0			
*Saa3* expression in lung			
Non-littering females	6	4	5
Littering females	11	12	13
F1			
Epididymal sperm parameters	11	11	12
DSP	10	11	12
SCSA	12	11	12
Plasma testosterone	11	12	11
F2			
Epididymal sperm parameters	11	10	14
DSP	11	10	14
Plasma testosterone	11	10	10
F2^a^			
Epididymal sperm parameters	11	10	8
DSP	11	9	5
Plasma testosterone	11	6	4
F3			
Epididymal sperm parameters	9	10	10
DSP	7	9	9
Plasma testosterone	6	6	7
F4			
Epididymal sperm parameters	7		6
DSP	7		6
Plasma testosterone	7		6

^a^F2 derived from the female germline

## Results

### Exposure and maternal pulmonary inflammation

Filter measurements demonstrated that the animals were exposed to a mean total suspended particle mass concentration of 4.79 ± 1.86 and 33.87 ± 14.77 mg/m^3^ for the low and high exposure, respectively. The control exposure was below the detection limit (LOD 0.08 mg/m^3^). The corresponding median particle concentrations in the exposure atmosphere with semi-interquartile deviation were 4 ± 4 cm^− 3^, 3.59 ± 2.48 × 10^5^ cm^− 3^ and 2.12 ± 0.69 × 10^6^ cm^− 3^ CB for the control, low, and high exposure level, respectively. The large variation for the low exposure primarily owes to the applied exposure regime, with the source being active for only two thirds of the exposure period. The measured aerosol number size distribution was found to be bi-modal, peaking at 100 and 300 nm. The cumulative deposited doses for the pulmonary, tracheobronchial and head region are presented in Table 2.

**Table 2:** Cumulative deposited doses (mg/kg bw) for pulmonary, tracheobronchial and head region

	Low	High
Pulmonary region	1.14	7
Tracheobronchial	1.25	7.12
Head	0.22	1.68

Cumulative deposited dose presented as mg/kg body weight

Pulmonary inflammation assessed in dams 11 and 28–29 days post-exposure showed no inflammation or acute phase response in terms of neutrophil influx or *Saa3* mRNA expression, respectively, as previously reported in Umezawa et al. 2018 [22]. Pregnant dams had normal weight gain during the exposure.

### Gestation and litter parameters

Gestation and litter parameters assessed for the F0 to F3 showed no changes in maternal weight gain during gestation and lactation, gestation length, number and loss of implantations, offspring weights, litter size and, sex ratio, for exposed females and offspring compared to control females and offspring (data not shown). During the breeding of the F2 to the F4 generations, almost all females cohabiting with a male delivered a litter. During breeding of the F2 generation, only two males, one each from the low and high exposure groups, did not produce a litter; during breeding of the F3 and F4 generations, one and two control males, respectively, did not produce a litter.

### Male-specific reproductive and fertility parameters

**Fig. 2:**
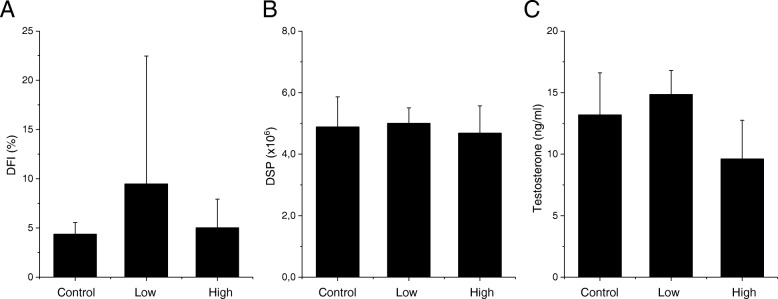
DNA fragmentation index, daily sperm production and testosterone concentrations in the F1 offspring. **a** DNA fragmentation index (%) measured in epididymal sperm, mean ± SD; **b** Daily sperm production (× 10^6^), measured in the testes, mean ± SD; **c** Testosterone concentration (ng/ml) measured in plasma, geometric mean ± geometric SD

**Fig. 3:**
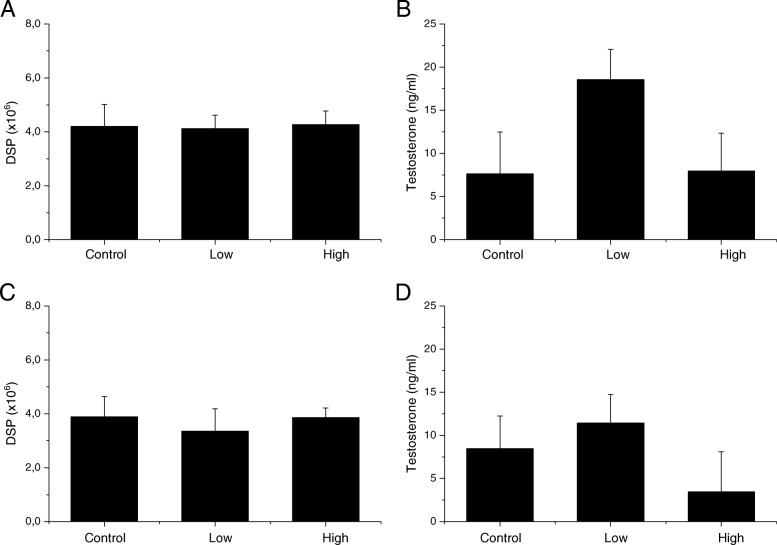
Daily sperm production and testosterone concentrations in the F2 offspring from the male and female germline. Daily sperm production (× 10^6^) measured in the testes, mean ± SD. Testosterone concentration (ng/ml) measured in plasma, geometric mean ± geometric SD. **a** DSP F2 male germline; **b** Testosterone F2 male germline; **c** DSP F3 female germline; **d** Testosterone F3 female germline

**Fig. 4:**
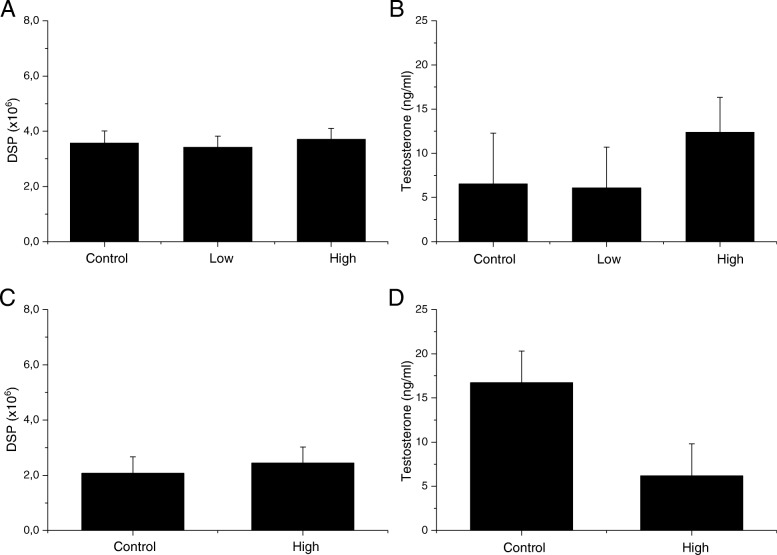
Daily sperm production and testosterone concentrations in the F3 and F4 offspring. Daily sperm production (× 10^6^) measured in the testes, mean ± SD. Testosterone concentration (ng/ml) measured in plasma, geometric mean ± geometric SD. **a** DSP F3; **b** Testosterone F3; **c** DSP F4; **d** Testosterone F4

There were no changes in body and reproductive organ weight and epididymal sperm parameters in any of the four generations from the male germ line and the F2 offspring from the female germline (Tables 3, 4, 5, 6, 7). There were also no changes in testicular sperm counts presented as DSP and plasma testosterone concentrations (Figs. 2, 3, 4). DNA fragmentation index in epididymal sperm, measured only for the F1, did not show any changes in maternally exposed animals compared to control offspring (Fig. 2). There was no difference in pregnancy index in exposed offspring compared to the control offspring.

**Table 3:** Body and organ weight and sperm parameters of the F1 offspring at 21 weeks of age

	First Generation (F1)		
	Control	Low	High
Body weight (g)	47.2 ± 5.8	47.5 ± 4.3	48.2 ± 5.02
Absolute left testes weight (mg)	107.6 ± 5.8	109.3 ± 7.3	104.9 ± 9.4
Relative testes weight (%)	0.2 ± 0.03	0.2 ± 0.02	0.2 ± 0.04
Absolute left epididymes weight (mg)	49.0 ± 3.1	48.7 ± 4.8	47.9 ± 8.3
Relative epididymes weight (%)	0.1 ± 0.02	0.1 ± 0.01	0.1 ± 0.02
Total epididymal sperm counts (× 10^6^)	33.6 ± 8.9	33.5 ± 8.1	34.0 ± 6.8
Total motility (%)	72.0 ± 12.8	72.2 ± 18.8	59.4 ± 12.9
Progressive motility (%)	21.1 ± 7.1	25.3 ± 7.7	21.1 ± 5.6

Data displayed as mean ± SD (*n* = 11–12)

**Table 4:** Body and organ weight and sperm parameters of the F2 offspring from the male germline at 11 weeks of age

	Second Generation Male Germline (F2)		
	Control	Low	High
Body weight (g)	40.2 ± 1.7	39.4 ± 2.4	41.7 ± 2.9
Absolute left testes weight (mg)	117.9 ± 8.3	114.7 ± 9.6	120.0 ± 7.3
Relative testes weight (%)	0.3 ± 0.02	0.3 ± 0.02	0.3 ± 0.03
Absolute left epididymes weight (mg)	50.6 ± 11.7	49.0 ± 9.3	43.8 ± 6.0
Relative epididymes weight (%)	0.1 ± 0.03	0.1 ± 0.02	0.1 ± 0.02
Total epididymal sperm counts (× 10^6^)	34.8 ± 6.2	36.0 ± 6.9	35.2 ± 5.3
Total motility (%)	65.1 ± 17.4	58.5 ± 14.9	67.1 ± 19.2
Progressive motility (%)	27.6 ± 9.2	25.4 ± 8.0	30.2 ± 8.8

Data displayed as mean ± SD (*n* = 10–14)

**Table 5:** Body and organ weight and sperm parameters of the F2 offspring from the female germline at 11 weeks of age

	Second Generation Female Germline (F3)		
	Control	Low	High
Absolute body weight (g)	43.4 ± 2.8	40.9 ± 2.6	40.8 ± 2.9
Absolute left testes weight (mg)	115.5 ± 9.0	111.8 ± 12.1	104.8 ± 21.8
Relative testes weight (%)	0.3 ± 0.03	0.3 ± 0.02	0.3 ± 0.05
Absolute left epididymes weight (mg)	46.6 ± 2.4	43.0 ± 4.9	46.0 ± 5.7
Relative epididymes weight (%)	0.1 ± 0.01	0.1 ± 0.01	0.1 ± 0.01
Total epididymal sperm counts (× 10^6^)	32.2 ± 5.0	35.5 ± 7.0	27.5 ± 8.4
Total motility (%)	65.3 ± 14.4	67.7 ± 16.9	75.0 ± 12.3
Progressive motility (%)	28.0 ± 4.9	30.8 ± 9.4	29.8 ± 8.7

Data displayed as mean ± SD (*n* = 8–11)

**Table 6:** Body and organ weight and sperm parameters of the F3 offspring at 12 weeks of age

	Third Generation (F3)		
	Control	Low	High
Absolute body weight (g)	42.2 ± 5.46	43.9 ± 3.94	41.5 ± 6.18
Absolute left testes weight (mg)	121.5 ± 18.1	113.4 ± 8.3	119.0 ± 11.0
Relative testes weight (%)	0.3 ± 0.1	0.3 ± 0.02	0.3 ± 0.04
Absolute left epididymes weight (mg)	44.6 ± 3.1	46.1 ± 3.1	39.6 ± 10.3
Relative epididymes weight (%)	0.1 ± 0.02	0.1 ± 0.01	0.1 ± 0.01
Total epididymal sperm counts (× 10^6^)	45.5 ± 13.6	42.1 ± 9.5	43.8 ± 8.1
Total motility (%)	44.9 ± 15.9	42.4 ± 9.1	41.9 ± 11.1
Progressive motility (%)	22.1 ± 10.5	20.5 ± 7.0	20.9 ± 7.2

Data displayed as mean ± SD (*n* = 9–10)

**Table 7:** Body and organ weight and sperm parameters of the F4 offspring at 8 weeks of age

	Fourth Generation (F4)	
	Control	High
Absolute body weight (g)	41.1 ± 1.4	39.4 ± 1.9
Absolute left testes weight (mg)	118.1 ± 10.1	106.8 ± 9.8
Relative testes weight (%)	0.3 ± 0.03	0.3 ± 0.01
Absolute left epididymes weight (mg)	43.2 ± 3.6	38.0 ± 9.2
Relative epididymes weight (%)	0.1 ± 0.01	0.1 ± 0.02
Total epididymal sperm counts (× 10^6^)	21.0 ± 9.7	27.1 ± 2.5
Total motility (%)	28.5 ± 6.6	35.0 ± 8.9
Progressive motility (%)	22.1 ± 4.8	28.1 ± 8.2

Data displayed as mean ± SD (*n* = 6–7)

## Discussion

Printex 90 consists of insoluble carbonaceous NPs with negligible content of metals, PAH and endotoxins [32]. These NPs are able to translocate from the lungs to the systemic circulation and deposit in secondary organs [15]. They are efficient generators of ROS, which in turn, may induce oxidative stress, inflammation and DNA damage [15, 33, 34]. Hence, maternal exposure to Printex 90 NPs may potentially affect fetal development directly as well as indirectly. We performed in utero exposure to Printex 90 by inhalation, the gold standard for pulmonary exposure, at occupationally relevant exposure levels to assess the effects of NPs on male-specific reproductive and fertility parameters in the offspring, and gestation and litter parameters in the time-mated dams, for four generations. We found no significant changes in male-specific reproductive and fertility parameters or gestation and litter parameters. Thus, we were unable to corroborate previous findings of reduced sperm counts in the testes of offspring following instillation of NPs during gestation [12, 13].

The present study followed up on an investigation from our laboratory of in utero exposure to instilled CB Printex 90 that reported decreased testicular sperm counts in the male offspring in the second generation of the male germline [13]. Instillation of 67 μg of Printex 90 on C57BL/6J mice on gestation day 7, 10, 15 and 18 caused sustained pulmonary inflammation in maternal lungs 26–27 days post instillation. Pulmonary inflammation in the dams caused by airway exposure to NPs is hypothesised to be a key mechanism of action for male-specific reproductive toxicity in the offspring. However, in the present study there was no indication of pulmonary inflammation in the maternal lungs when assessed 11 and 28–29 days post-exposure in terms of BAL cell counts and *Saa3* expression. The different lung responses observed after instillation versus inhalation exposure cannot be ascribed to variation in exposure levels as these were assumed to be similar between the two studies: the cumulative dose of 268 μg of instilled Printex 90 in the C57BL/6J mice could roughly correspond to an inhaled cumulative dose of 280 μg for an NMRI mouse weighing an average weight of ~ 40 g at the high exposure level of Printex 90 in this study (estimated using the calculated deposited dose in mg/kg). We have previously observed that animals instilled with vehicle control had similar pulmonary inflammation in terms of neutrophil influx and *Saa3* expression as unhandled controls; therefore, the effects observed in the particle exposed groups cannot be attributed to the instillation procedure itself (*Saa3* expression data not published) [30]. Likewise, in our previous study, we used nanopure water as instillation vehicle to avoid vehicle effects [33]. However, at similar exposure levels, intratracheally instilled NPs induce a stronger inflammatory response in the lungs than inhaled NPs [34]. Still, since CB-dependent inflammation decreases over time [35, 36], we cannot rule out that pulmonary inflammation may have occurred at an earlier time point.

There were no differences in litter and gestation parameters between F1 control and F1 offspring from CB dams exposed by instillation or inhalation. However, the F2 offspring from the male germline of instilled CB dams had statistically significantly lower testicular sperm content per gram of testis following instillation. Lower DSP was also reported in the same F2 offspring, but it was not statistically different from controls. In the present study, testicular sperm content per gram of testis (data not shown) and DSP were unaffected in the F2 offspring, and in the subsequent generations. One major difference between the two studies is that in Kyjovska et al. [13] C57BL6/6J mice were used for the F0 and F1 generation and two different strains of mice (C57BL/6J and CBA/J) were used to produce the F2 offspring, making them heterozygotic, whereas in the present study NMRI mice were used throughout. The use of different strains may restrict the comparison of the two studies since reproductive factors, such as semen quality and susceptibility to toxic insult, are strain specific [37, 38]. Furthermore, the Kyjovska et al. [13] study is limited in the fact that only organ weights and testicular sperm counts were assessed. Adequate testing for reproductive toxicity requires that a broad spectrum of potential effects is covered [39]. In the present study, we report several male-specific parameters (i.e., organ weights, several sperm parameters, hormone levels, etc.) and gestation and litter parameters, and we do so for four generations. Several of the applied methods have been previously validated in our laboratory, including the assessment of DSP [13] and the testosterone assay (unpublished data). In addition, the use of a computer assisted sperm analysis system provides automated and accurate measurements of each individual sperm and eliminates bias from manual estimates [40].

Our study has some limitations. Although, an a priori analysis indicated that a group size of 15 was needed to detect a statistically significant difference in the DSP, due to the decrease in mating couples from F1 and onwards, it was challenging to maintain the required sample sizes and statistical power was consequently lowered especially in the later generations. In addition, in the Kyjovska et al. [13] study the experimental groups were only statistically significantly different when testicular sperm counts were normalized to testicular weight. The latter may be difficult to interpret since testes organ weight is independent of sperm production rate in some rodent species e.g. Fischer 344 rats [41]. Nevertheless, intratracheal instillation of pregnant ICR mice to 0.2 mg/mouse of a 14 nm CB on gestation day 7 and 14 induced testicular effects in offspring from exposed dams. The authors reported partial vacuolation of the seminiferous tubules, reduced cellular adhesion of the seminiferous epithelium, as well as reduced DSP in the offspring, at 5, 10 and 15 weeks of age. However, there was no difference in body and organ weight, testosterone concentration, epididymal sperm concentration, motility, gestation length, litter size, fertility or gender ratio [12]. In comparison to Kyjovska et al. [13] and the present study, the cumulative dose was considerably higher (400 μg) and the 14 nm CB particles were dispersed in saline solution with 0.05% Tween 80. It has been previously argued that the use of dispersants appears to influence the bioavailability of nanoparticles and toxicity on the male reproductive system following pulmonary exposure [30].

Instillation studies are useful because mechanistic effects of known doses of NPs in the lungs can be investigated. However, this route of administration presents with several limitations, one of which is that it is not a relevant exposure scenario for humans. Inhalation exposure, on the other hand, provides the opportunity to study relevant exposure levels and scenarios and is therefore considered the golden standard in risk assessment of airborne compounds. In utero exposure to 20 mg/m^3^ of diesel exhaust particle (SRM2975) for 1 h/day on gestation days 7 to 19 via whole body inhalation was associated with reduced DSP (reported as the median) in C57BL/6J mice [11]. However, there was no difference in body and organ weight, anogenital distance, testosterone and estradiol plasma concentrations. Also, no endocrine disruption activity was observed in the offspring from exposed dams (i.e., gene regulation of the androgen receptor, anti-Müllerian hormone, estrogen receptor-α, estrogen receptor-β, follicle-stimulating hormone receptor, insulin-like growth factor 3, luteinizing hormone receptor, and aromatase in testes were not significantly altered) [11]. The exposure in this and the present study compare, except for the type of particle. Diesel exhausts particles, unlike CB, have a high level of adhered compounds such as PAHs and heavy metals that are thought to leach from the particles and elicit added toxic effects [42].

Based on the above, it is apparent that several studies investigating in utero exposure to carbonaceous NPs and reproductive effects find reduced DSP but with very little or no additional reproductive effects. The enumeration of testicular sperm heads is not a novel method, yet it is a quantitative method that is simple, reproducible and sensitive and therefore it is a common assay to apply in reproductive toxicity [13, 41]. The principle is simple: mature sperm heads are resistant to homogenization due to their highly condensed and cross-linked nucleoproteins, when the testis is homogenized all other cell types are destroyed leaving the sperm heads intact and visible for counting under a microscope in a counting chamber. However, this method has its limitations as a stand-alone result since it cannot provide information on the mechanisms or the specific testicular cell types that are sensitive to the toxic insult. In addition, the results may be hard to interpret, i.e., decreased sperm counts are not necessarily a reflection of direct toxicity to spermatogenic cells (toxicity may occur on other testicular cells i.e. Sertoli and Leydig cells); abnormal accumulation of spermatids in the testes may occur due to disturbed spermatogenesis and spermiation causing increased testicular sperm counts and even normal sperm counts does not necessarily mean absence of toxic insult [41]. Furthermore, it is evident that researchers apply different protocols, calculations and statistical analysis for the DSP which make it challenging to compare results.

We recently assessed the effect on male reproductive parameters in adult NMRI mice following a seven-week airway exposure to carbonaceous NPs, initiated at 8 weeks of age [30]. No effects on male reproductive parameters were found in spite of the substantial pulmonary inflammation induced by the four different carbonaceous NPs used, including diesel exhaust NPs and CB Printex 90 [30]. We concluded that the inflammation alone caused by the NPs, at least in adult mice, may not be necessarily an important determinant of male reproductive toxicity. It was recently shown that CB NP-dependent DNA damage in liver tissue following pulmonary exposure was not caused indirectly by pulmonary inflammation, but was likely caused by surface-mediated generation of ROS of translocated CB NPs [15]. Perhaps the direct effects of translocated NPs and adhered compounds may be of higher significance for male reproductive toxicity than the indirect effects of inflammatory mediators. With regards to instillations, very similar rates of translocation from lung to liver were found following inhalation and intratracheal instillation of nanosized TiO_2_ particles [43]. If a greater fraction of instilled NPs deposits deeper into the lungs and are cleared more slowly; and particle induced inflammation increases permeability of the blood-alveolar membrane, accordingly intratracheal instillations may lead to higher levels of particle translocation [44, 45]. If so, this may explain the pronounced effects in the Yoshida et al. 2010 instillation study [12]. We have previously shown that in utero exposure to the diesel exhaust particle NIST2975 at similar dose levels (20 mg/m^3^ for 1 h/day) induced DNA instability in terms of increased microsatellite instability in the male germline DNA [46–48]. In adulthood, these effects have been shown to depend on the particulate fraction of the emissions, since HEPA filtration reduced the microsatellite instability [49]. If it is in fact the direct effect of the particles, then arguably the proportion of translocated NPs from the lungs to the fetus may not be sufficient to elicit a detectable change in the male reproductive system. Translocation of lung deposited NPs to the systemic circulation is low (1–2%) and the majority of translocated NPs will accumulate in secondary target organs such as the liver, spleen and the placenta. In fact, studies show that the translocation across the placenta is very low (0.005–0.018%) hence resulting in very limited translocation to the fetus [17, 50, 51]. Gestational or developmental toxicity on the male reproductive system were not observed even when persistent lung inflammation was present [34]. Thus, lung inflammation may not be responsible of fetal developmental toxicity.

Even if the findings of unaltered sperm parameters in the mouse offspring are reassuring, it would be premature to conclude that exposure to carbon particles does not pose a risk to male reproduction in humans. In the present study, exposure was initiated at GD 4, and by using the same experimental setup, developmental neurotoxicity was observed in offspring of the F1 generation [22]. Human exposure, occupationally or in private life, would be expected to start prior to fertilization. Hence, particles could have accumulated in lung tissue and possibly have translocated systemically and be present in the reproductive organs, when pregnancy was established. In addition, differences in human and rodent placentation may potentially lead to larger translocation of particles in humans compared to mice, at the later stages of pregnancy and male reproductive function in humans may be more vulnerable to toxicological insult than that of rodents [16, 52].

## Conclusion

The present study followed up earlier investigations of maternal instillation exposure to CB Printex 90, reporting decreased sperm counts in F1 and F2 males [12, 13]. Our study showed that maternal inhalation exposure to CB NPs at occupational exposure levels did not affect male-specific reproductive and fertility parameters in four generations of male offspring, nor were gestation and litter parameters affected.

## Abbreviations

BAL: Bronchoalveolar lavage
CB: Carbon Black
DSP: Daily sperm production
F1: First generation
F2: Second generation
F3: Third generation
F4: Fourth generation
NPs: Nanoparticles
ROS: Reactive oxygen species
SCSA: Sperm Chromatin Structure Assay
PND: Postnatal day

## References

[1] Carlsen E, Giwercman A, Keiding N, Skakkebaek NE (1992). Evidence for decreasing quality of semen during past 50 years. BMJ.

[2] Levine H, Jørgensen N, Martino-Andrade A, Mendiola J, Weksler-Derri D, Mindlis I, Pinotti R, Swan SH (2017). Temporal trends in sperm count: a systematic review and meta-regression analysis. Hum Reprod Update.

[3] Jensen TK, Carlsen E, Jørgensen N, Berthelsen JG, Keiding N, Christensen K, Petersen JH, Knudsen LB, Skakkebæk NE (2002). Poor semen quality may contribute to recent decline in fertility rates. Hum Reprod.

[4] Priskorn L, Nordkap L, Bang AK, Krause M, Holmboe SA, Egeberg Palme DL (2018). Average sperm count remains unchanged despite reduction in maternal smoking: results from a large cross-sectional study with annual investigations over 21 years. Hum Reprod.

[5] Bonde JP (2010). Male reproductive organs are at risk from environmental hazards. Asian J Androl.

[6] Ema M, Hougaard KS, Kishimoto A, Honda K (2016). Reproductive and developmental toxicity of carbon-based nanomaterials: a literature review. Nanotox.

[7] Stillerman KP, Mattison DR, Giudice LC, Woodruff TJ (2008). Environmental exposures and adverse pregnancy outcomes: a review of the science. Reprod Sci.

[8] Håkonsen L, Ernst A, Ramlau-Hansen CH (2014). Maternal cigarette smoking during pregnancy and reproductive health in children: a review of epidemiological studies. Asian J Androl.

[9] Bonde JP, Flachs EM, Rimborg S, Glazer CH, Giwercman A, Ramlau-Hansen CH, Hougaard KS (2017). The epidemiologic evidence linking prenatal and postnatal exposure to endocrine disrupting chemicals with male reproductive disorders: a systematic review and meta-analysis. Hum Reprod Update.

[10] Meier MJ, O’Brien JM, Beal MA, Allen B, Yauk CL, Marchetti F (2017). In utero exposure to benzo(a)pyrene increases mutation burden in the soma and sperm of adult mice. Environ Health Perspect.

[11] Hemmingsen JG, Hougaard KS, Talsness C, Wellejus A, Loft S, Wallin H, Møller P (2009). Prenatal exposure to diesel exhaust particles and effect on the male reproductive system in mice. Toxicology.

[12] Yoshida S, Hiyoshi K, Oshio S, Takano H, Takeda K, Ichinose T (2010). Effects of fetal exposure to carbon nanoparticles on reproductive function in male offspring. Fertil Steril.

[13] Kyjovska ZO, Boisen AMZ, Jackson P, Wallin H, Vogel U, Hougaard KS (2013). Daily sperm production: application in studies of prenatal exposure to nanoparticles in mice. Reprod Toxicol.

[14] Klengel T, Dias BG, Ressler J (2016). Models of intergenerational and transgenerational transmission of risk for psychopathology in mice. Neuropsychopharmacology.

[15] Modrzynska J, Berthing T, Ravn-Haren G, Jacobsen NR, Weydahl IK, Loeschner K, Mortensen A, Thoustrup AS, Vogel U (2018). Primary genotoxicity in the liver following pulmonary exposure to carbon black nanoparticles in mice. Part Fiber Toxicol.

[16] Hougaard KS, Campagnolo L, Chavatte-Palmer P, Tarrade A, Rousseau-Ralliard D, Valentino S (2015). A perspective on the developmental toxicity of inhaled nanoparticles. Reprod Toxicol.

[17] Campagnolo L, Massimiani M, Vecchione L, Piccirilli D, Toschi N, Magrini A (2017). Silver nanoparticles inhaled during pregnancy reach and affect the placenta and the foetus. Nanotox.

[18] Semmler-Behnke M, Lipka J, Wenk A, Hirn S, Schäffler M, Tian F, Schmid G (2014). Size dependent translocation and fetal accumulation of gold nanoparticles from maternal blood in the rat. Part Fibre Toxicol.

[19] Jacobsen NR, Pojana G, White P, Møller P, Cohn CA, Korsholm KS, Vogel U, Marcomini A, Loft S, Wallin K (2008). Genotoxicity, cytotoxicity, and reactive oxygen species induced by single-walled carbon nanotubes and C(60) fullerenes in the FE1-Mutatrade mark mouse lung epithelial cells. Environ Mol Mutagen.

[20] Karsch FJ, Battaglia DF, Breen KM, Debus N, Harris TG (2002). Mechanisms for ovarian cycle disruption by immune/inflammatory stress. Stress..

[21] Miranda A, Sousa N (2018). Maternal hormonal milieu influence on fetal brain development. Brain Behav.

[22] Umezawa M, Onoda A, Korshunova I, Jensen ACØ, Koponen IK, Jensen KA, Khodosevich K, Vogel U, Hougaard KS (2018). Maternal inhalation of carbon black nanoparticles induces neurodevelopmental changes in mouse offspring. Part Fiber Toxicol.

[23] Bourdon JA, Saber AT, Jacobsen NR, Jensen KA, Madsen AM, Lamson JS, Wallin H (2012). Carbon black nanoparticle instillation induces sustained inflammation and genotoxicity in mouse lung and liver. Part and Fibre Toxicol.

[24] Jackson P, Kling K, Jensen KA, Clausen PA, Madsen AM, Wallin H, Vogel U (2015). Characterization of genotoxic response to15 multiwalled carbon nanotubes with variable physicochemical properties including surface Functionalizations in the FE1-Muta(TM) mouse lung epithelial cell line. Environ Mol Mutagen.

[25] The Danish Working Environment Authority. At-vejledning. Stoffer og Materiale - C.0.1. Grænseværdier for stoffer og materialer. 2017;1–84.

[26] Bourdon JA, Saber AT, Jacobsen NR, Jensen KA, Madsen AM, Lamson JS, Vogel U (2012). Carbon black nanoparticle instillation induces sustained inflammation and genotoxicity in mouse lung and liver. Part Fibre Toxicol.

[27] Anjilvel and Asgharian (1994). A multiple-path model of particle deposition in the rat lung. Fundam Appl Toxicol.

[28] Asgharian B, Price OT, Oldham M, Chen LC, Saunders EL, Gordon T (2014). Computational modeling of nanoscale and microscale particle deposition, retention and dosimetry in the mouse respiratory tract. Inhal Toxicol.

[29] Miller FJ, Asgharian B, Schroeter JD, Price O (2014). Improvements and additions to the multiple path particle dosimetry model. J Aerosol Sci.

[30] Skovmand A, Lauvås AJ, Christensen P, Vogel U, Hougaard KS, Goericke-Pesch S (2018). Pulmonary exposure to carbonaceous nanomaterials and sperm quality. Part Fibre Toxicol.

[31] Maurice C, O’Brien JM, Yauk CL, Marchetti F (2018). Integration of sperm DNA damage assessment into OECD test guidelines for genotoxicity testing using the MutaMouse model. Toxicol Appl Pharmacol.

[32] Saber AT, Jensen KA, Jacobsen NR, Birkedal R, Mikkelsen L, Møller P, Loft S, Wallin H, Vogel U (2012). Inflammatory and genotoxic effects of nanoparticles designed for inclusion in paints and lacquers. Nanotox.

[33] Hadrup N, Bengtson S, Jacobsen NR, Jackson P, Nocun M, Saber AT (2017). Influence of dispersion medium on nanomaterial-induced pulmonary inflammation and DNA strand breaks: investigation of carbon black, carbon nanotubes and three titanium dioxide nanoparticles. Mutagenesis.

[34] Jackson P, Hougaard KS, Boisen AMZ, Jacobsen NR, Jensen KA, Møller P (2012). Pulmonary exposure to carbon black by inhalation or instillation in pregnant mice: effects on liver DNA strand breaks in dams and offspring. Nanotox..

[35] Jacobsen NR, Møller P, Jensen KA, Vogel U, Ladefoged O, Loft S (2009). Lung inflammation and genotoxicity following pulmonary exposure to nanoparticles in ApoE−/−mice. Part Fibre Toxicol.

[36] Husain M, Kyjovska ZO, Bourdon-Lacombe J, Saber AT, Jensen KA, Jacobsen NR (2015). Carbon black nanoparticles induce biphasic gene expression changes associated with inflammatory responses in the lungs of C57BL/6 mice following a single intratracheal instillation. Toxicol Appl Pharmacol.

[37] Krzanowska H (1981). Sperm head abnormalities in relation to the age and strain of mice. J Reprod Fertil.

[38] Thurston LM, Watson PF, Holt WV (2002). Semen cryopreservation: a genetic explanation for species and individual variation?. CryoLetters.

[39] Kimmel GL, Clegg ED, Crisp TM. Reproductive toxicity testing. In: Witorsch RJ, editor. Reproductive Toxicology. 2nd ed. New York: Raven Press; 1995. p.75–98.

[40] Amann RP, Waberski D (2014). Computer-assisted sperm analysis (CASA): capabilities and potential developments. Theriogenology.

[41] Chapin RE, Heindel JJ. Methods in Toxicology. California: Academic Press, Inc.; 1993;3.

[42] Kyjovska ZO, Jacobsen NR, Saber AT, Bengtson S, Jackson P, Wallin H, Vogel U (2015). DNA strand breaks, acute phase response and inflammation following pulmonary exposure by instillation to the diesel exhaust particle NIST1650b in mice. Mutagenesis.

[43] Modrzynska J, Berthing T, Ravn-Haren G, Kling K, Mortensen A, Rasmussen RR, Larsen EH, Saber AT, Vogel U, Loeschner K (2018). In vivo-induced size transformation of cerium oxide nanoparticles in both lung and liver does not affect long-term hepatic accumulation following pulmonary exposure. PLoS One.

[44] Chen J, Tan M, Nemmar A, Song W, Dong M, Zhang G, Li Y (2006). Quantification of extrapulmonary translocation of intratracheal-instilled particles in vivo in rats: effect of lipopolysaccharide. Toxicology..

[45] Meiring J, Borm PJA, Bagate K, Semmler M, Seitz J, Takenaka S, Kreyling WG (2005). The influence of hydrogen peroxide and histamine on lung permeability and translocation of iridium nanoparticles in the isolated perfused rat lung. Part Fibre Toxicol.

[46] Boisen AMZ, Shipley T, Jackson P, Hougaard KS, Wallin H (2012). NanoTIO 2 (UV-titan) does not induce ESTR mutations in the germline of prenatally exposed female mice. Part Fibre Toxicol.

[47] Ritz C, Ruminski W, Hougaard KS, Wallin H, Vogel U, Yauk CL (2011). Germline mutation rates in mice following in utero exposure to diesel exhaust particles by maternal inhalation. Mutat Res.

[48] Boisen AMZ, Shipley T, Jackson P, Wallin H, Nellemann C, Vogel U (2013). In utero exposure to nanosized carbon black (Printex90) does not induce tandem repeat mutations in female murine germ cells. Reprod Toxicol.

[49] Yauk C, Polyzos A, Rowan-Carroll A, Somers CM, Godschalk RW, Van Schooten FJ (2008). Germ-line mutations, DNA damage, and global hypermethylation in mice exposed to particulate air pollution in an urban/industrial location. PNAS.

[50] Takahashi S, Matsuoka O (1981). Cross placental transfer of 198Au-colloid in near term rats. J Radiat Res.

[51] Geiser M, Kreyling WG (2010). Deposition and biokinetics of inhaled nanoparticles. Part Fibre Toxicol.

[52] Working PK (1988). Male reproductive toxicology: comparison of the human to animal models. Environ Health Perspect.

